# Sonographic Imaging of the Median Nerve in Elderly Patients With Motor Disability

**DOI:** 10.7759/cureus.93529

**Published:** 2025-09-29

**Authors:** Kholoud Sandougah, Mohamed Bedewi, Saeed M Alamri, Nawaf Alzain, Abdulrahman A Alharthi, Mamdouh A Kotb, Mohammed A Zayed, Mohamed Sherif Elsharkawy, Saleh M Alfawaz, Husain Alturkistani, Elsayed A Beheri, Rasha Ali

**Affiliations:** 1 Internal Medicine, College of Medicine, Imam Mohammad Ibn Saud Islamic University, Riyadh, SAU; 2 Department of Internal Medicine, College of Medicine, Prince Sattam bin Abdulaziz University, Al-Kharj, SAU; 3 Medical Rehabilitation, King Saud University Medical City, Riyadh, SAU; 4 Radiology, King Saud University Medical City, Riyadh, SAU; 5 Radiology and Medical Imaging, King Saud University Medical City, Riyadh, SAU; 6 Department of Family Medicine, Prince Sattam bin Abdulaziz University Hospital, Al-Kharj, SAU; 7 Department of Radiology and Medical Imaging, College of Medicine, King Saud University, Riyadh, SAU; 8 Radiology, King Saud University Medical City, Riaydh, SAU; 9 Training Sports and Kinesiology, College of Sports Science, Arish University, Arish, EGY

**Keywords:** cross-sectional area, disabled, elderly, median nerve, peripheral nerves, ultrasound

## Abstract

Objective

The aim of this study is to examine the median nerve (MN) cross-sectional area (CSA) by ultrasound at the level of the carpal tunnel in elderly patients with motor disability.

Materials and methods

The study sample included 124 median nerves in 62 participants; 16 of them were disabled elderly patients (13 males, three females), mean age 66.6, mean height 167.1 cm, mean weight 83.9 kg, mean BMI 29.9. Twenty-three were young controls (six males, 17 females), mean age 48.4, mean height 154.9 cm, mean weight 79.8 kg, mean BMI 32.7, and 23 were elderly nondisabled participants (13 males, 10 females), mean age 63.6, mean height 161.9 cm, mean weight 80.56 kg, mean BMI 30.88.

Results

The mean CSA of the MN in the elderly disabled group was 11 mm^2^. The mean CSA of the young control group MN was 10.45 mm^2^. The mean CSA of the MN in the elderly nondisabled group (both diabetic and nondiabetic) was 13.04 mm^2^. The mean CSA of the MN in the elderly diabetic nondisabled group was 14.5 mm^2^. The mean CSA of the MN in the elderly (nondiabetic) nondisabled group was 11.65 mm^2^.

Conclusion

Our study suggests that nerve ultrasound could be a helpful tool for assessment of the median nerve in elderly patients with motor disability.* *

## Introduction

Musculoskeletal disorders with disability are reported by the WHO to be one of the major chronic conditions of the elderly population. More than 20% of the elderly population have limited daily life activities resulting from disability, likely due to musculoskeletal injury [1-4]. The disabled elderly population faces long-term physical, sensorimotor, and mental difficulties which could cause partial or full restriction of social engagement [5-7]. Peripheral nerve disorders are considered one of the important causes of disability in the elderly population. Choosing the most suitable treatment is not a simple task in this set of diseases [8-12]. Peripheral nerve ultrasound is now used for two decades in different types of disabled patients during the rehabilitation period [13]. Among the advantages of ultrasound are cheap price, lack of ionizing radiation, portability, dynamic nature, and possibility of examination of the contralateral side at the same session. Ultrasound can also easily be used for patients with prosthetic devices [3]. The ease of examination with handheld ultrasound devices could be of help in elderly disabled patients, especially in cases where repeat examination for follow-up is needed. The cross-sectional area (CSA) is considered the main sonographic parameter for assessment of the peripheral nerves. The aim of this study is to assess the CSA of the median nerve (MN) at the level of the carpal tunnel in elderly patients with motor disability (DE).

## Materials and methods

Participants

After institutional review board approval from Al Imam Mohammed Ibn Saud Islamic University (approval HAPO-01-R-011), participants of the study were recruited between February 2025 and May 2025, and written consent was obtained. Sixteen elderly patients with different types of motor disability were recruited to a university hospital. Inclusion criteria included elderly patients at least 60 years old with DE. A second group included elderly patients at least 60 years old without motor disability, nondisabled elderly (NDE), who were subdivided into diabetic (type II) and nondiabetic. Nerve conduction study was performed on a quarter of the participants. The third group included healthy subjects below 60 years old. For each participant, data including age, sex, BMI, weight, and height were recorded.

Technique

**Figure 1:**
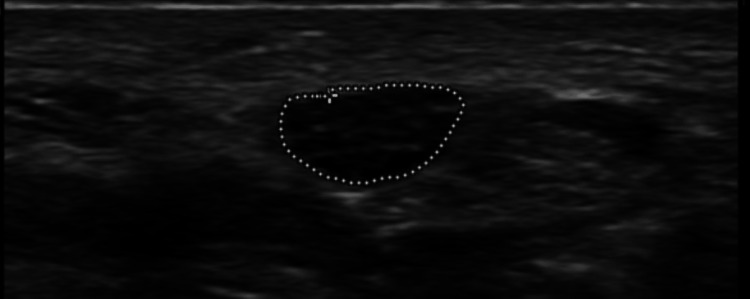
Short axis scan of an enlarged median nerve at the wrist; cross-sectional area (CSA) = 17.5 mm2

We used L12-5 and L18-5 MHz linear transducers (EPIQ 7 version 1.5 ultrasound system; Philips Ultrasound, Inc., Bothell, WA, USA). All participants were scanned separately by a radiologist (M.B.) with 13 years of experience in neuromuscular ultrasonography, and revised by a neurologist (M.K.) with seven years of experience in neuromuscular ultrasound. Each nerve was scanned three times, with the ultrasound probe removed from the skin in between. The MN was scanned bilaterally at the inlet of the carpal tunnel and identified by its fascicular honeycomb pattern. The CSA of the MN was measured in mm2 in short axis view inside the hyperechoic epineurium using the tracer method. Minimal pressure was exerted on the probe to optimize image quality and the transducer was strictly perpendicular to avoid anisotropy (Figure 1).

Statistical analysis

Statistical analysis was performed using SPSS version 27 software (IBM Corp., Armonk, NY, USA). All data were presented as mean standard deviation (SD) and range. The Shapiro-Wilk test was used to assess the normality of the data distribution. The correlations between the CSA of the scanned nerves, age, weight, height, and BMI were evaluated using Pearson’s correlation coefficient (r). A P value of <.05 was considered significant.

## Results

The study sample included 124 median nerves in 62 participants. Sixteen of them were disabled elderly patients (13 males, three females), mean age 66.6 ± 4.16, mean height 167.1 cm ± 9.2, mean weight 83.9 kg ± 13.1, mean BMI 29.9 ± 4.2. Twenty-three were young controls (six males, 17 females), mean age 48.4 ± 5.8, mean height 154.9 cm ± 6.48, mean weight 79.8 kg ± 13.1, mean BMI 32.7 ± 4.2, and 23 were elderly nondisabled participants (13 males, 10 females), mean age 63.6 ± 4.93, mean height 161.9 cm ± 9.11, mean weight 80.56 kg ± 15.88, mean BMI 30.88 ± 5.3 (Table 1). The intra-observer reliability calculations resulted in an overall intra-class correlation coefficient of 0.88 and moderate to good agreement between the examiners. The mean CSA of the MN in the elderly disabled group was 11 mm2 ± 3.18. The mean CSA of the young control group MN was 10.45 mm2 ± 3.76 SD. The mean CSA of the MN in the elderly nondisabled group (both diabetic and nondiabetic) was 13.04 mm2 ± 3.09. The mean CSA of the MN in the elderly diabetic nondisabled group was 14.5 mm2 ± 2.49. The mean CSA of the MN in the elderly (nondiabetic) nondisabled group was 11.65 mm2 ± 1.1 (Table 2). The MN CSA in the NDE group showed moderate negative correlation with BMI (p = 0.104). Otherwise, all three groups showed no significant statistical correlation with all other demographic factors (Table 3). 

**Table 1:** Demographic data of study participants DM = type II diabetes mellitus

Variable	Control Aged more than 60 (n = 23)		Non-DM (n = 10)		DM (n = 13)		Control less than 60 (n = 23)		Elderly Disabled (n = 16)		
	N	%	N	%	N	%	N	%	N	%	
Gender											
Male	13	56.52%	7	70%	6	46.15%	6	26.08%	13	81.25%	
Female	10	43.47%	3	30%	7	53.84%	17	73.91	3	18.75%	
	Mean± SD		Mean± SD		Mean± SD		Mean± SD		Mean± SD		P value
Age (years)	(63.60±4.93)		(62.30±3.27)		(65.30±6.29)		(48.43±5.85)		(66.62±4.16)		<001
Height (cm)	(161.91±9.11)		(161.76±9.99)		(162.10±8.34)		(154.93±6.48)		(167.12±9.20)		<001
Weight (kg)	(80.56±15.88)		(86.43±16.87)		(72.95±11.07)		(79.80±14.64)		(83.91±14.15)		.306
Body mass index (kg/m²)	(30.88±5.36)		(33.23±5.07)		(27.83±4.19)		(32.70±5.73)		(29.98±4.25)		.211

**Table 2:** The mean median nerve CSA measured in square millimeter in different age groups CSA = cross-sectional area, MN = median nerve, DM = type II diabetes mellitus, SD = standard deviation

Variable	Control elderly non disabled (n = 23)	Non-DM (n = 10)	DM (n = 13)	Control young (n = 23)	Elderly Disabled (n = 16)	
	Mean± SD	Mean± SD	Mean± SD	Mean± SD	Mean± SD	P value
MN CSA	(13.04±3.09)	(11.65±1.10)	(14.50±2.49)	(10.45±3.76)	(11.06±3.18)	0.981

**Table 3:** Correlation between median nerve CSA, age, height, weight, and BMI in the disabled elderly group CSA = cross-sectional area, BMI = body mass index.

		Median Nerve	Age	Height	Weight	BMI
Median Nerve	Pearson Correlation	1	0.132	-0.137	0.171	0.325
	Sig. (2-tailed)		0.627	0.612	0.526	0.220

## Discussion

In our study, the MN CSA in the disabled elderly group (11 mm2) was larger compared to the control young population group (10.45 mm2), and was also increased compared to other studies in the literature like Kerasnoudis et al. (8.75 mm2) [14], Fisse et al. (8.3 mm2) [15], Won et al. (8.32 mm2) [16], and Qrimli et al. (10 mm2) [17]. The MN CSA in the elderly disabled group was relatively smaller than the elderly non-disabled non-diabetic group (11.65 mm2); however, the elderly disabled group showed considerably smaller CSA than the mean of both diabetic and nondiabetic elderly non-disabled groups together (13.04 mm2), and even smaller than the elderly diabetic group alone (14.5 mm2). Our results suggest an increased CSA of the median nerve in elderly patients with motor disability in comparison to control groups, with suggested exempt of diabetic patients. Increased CSA of the peripheral nerves, especially the median nerve, is reported in literature to be related to diabetes mellitus (DM) with or without polyneuropathy. Essentially, the relation between DM and aging is complex and often described as synergistic. On a molecular basis, the underlying pathophysiology of both DM and aging seems to run parallel. Actually the aging process itself should be dealt with as a risk factor for insulin resistance and the resultant morphologic abnormalities and functional impairment could be due to changes in the microvascularity [18-20]. 

Limitations

This study has several limitations. First is the small sample size. Second, the sample of the disabled elderly group included different causes of motor disability like cerebrovascular accidents, chronic peripheral nerve disease, poliomyelitis, spinal fusion, and scoliosis. This variability with different scopes is associated with uneven distribution of muscle and nerve involvement. Third, examining the median nerve was challenging in three cases of disability post cerebrovascular accident due to uncooperative patients. Fourth, nerve conduction studies were only done for a sample study participant. Fifth, only cases of motor disabilities were included. Further studies involving different categories of disability are encouraged, especially those related to sensorimotor causes.

## Conclusions

In conclusion, our study suggests that nerve ultrasound could be a helpful tool for assessment of the median nerve in elderly patients with motor disability.
