# Correction: Sonographic Imaging of the Median Nerve in Elderly Patients With Motor Disability

**DOI:** 10.7759/cureus.c408

**Published:** 2026-02-26

**Authors:** Kholoud Sandougah, Mohamed Bedewi, Saeed M Alamri, Nawaf Alzain, Abdulrahman A Alharthi, Mamdouh A Kotb, Mohammed A Zayed, Mohamed Sherif Elsharkawy, Saleh M Alfawaz, Husain Alturkistani, Elsayed A Beheri, Rasha Ali

**Affiliations:** 1 Internal Medicine, College of Medicine, Imam Mohammad Ibn Saud Islamic University, Riyadh, SAU; 2 Department of Internal Medicine, College of Medicine, Prince Sattam bin Abdulaziz University, Al-Kharj, SAU; 3 Medical Rehabilitation, King Saud University Medical City, Riyadh, SAU; 4 Radiology, King Saud University Medical City, Riyadh, SAU; 5 Radiology and Medical Imaging, King Saud University Medical City, Riyadh, SAU; 6 Department of Family Medicine, Prince Sattam bin Abdulaziz University Hospital, Al-Kharj, SAU; 7 Department of Radiology and Medical Imaging, College of Medicine, King Saud University, Riyadh, SAU; 8 Radiology, King Saud University Medical City, Riaydh, SAU; 9 Training Sports and Kinesiology, College of Sports Science, Arish University, Arish, EGY

This article has been corrected to revise the affiliation of Husain Alturkistani.

